# Phospholipase Cγ2 Signaling Cascade Contribute to the Antiplatelet Effect of Notoginsenoside Fc

**DOI:** 10.3389/fphar.2018.01293

**Published:** 2018-11-06

**Authors:** Yingqiu Liu, Tianyi Liu, Kevin Ding, Zengyuan Liu, Yuanyuan Li, Taotao He, Weimin Zhang, Yunpeng Fan, Wuren Ma, Li Cui, Xiaoping Song

**Affiliations:** ^1^Laboratory of Traditional Chinese Veterinary Medicine, College of Veterinary Medicine, Northwest A&F University, Yangling, China; ^2^Department of Neurosurgery, The First Hospital of Jilin University, Changchun, China; ^3^University of Illinois at Urbana–Champaign, Urbana, IL, United States; ^4^Department of Neurosciences, University of California, San Diego School of Medicine, La Jolla, CA, United States

**Keywords:** *Panax notoginseng*, Notoginsenoside Fc, platelet aggregation, antiplatelet effect, thrombosis, phospholipase Cγ2

## Abstract

**Scope:** Bleeding, the main drawback of clinically used chemical anti-thrombotic drug is resulted from the unidirectional suppression of platelet activity. Therefore, dual-directional regulatory effect on platelet is the main preponderance of *Panax notoginseng* over these drugs. The dual-directional regulatory effect should be ascribed to the resourceful *Panax notoginseng* saponins (PNS). Clarifying the mechanism of main PNS in both inhibiting and promoting platelet aggregation will give a full outlook for the dual-directional regulatory effect. The present study is aimed at explaining the mechanism of Notoginsenoside Fc (Fc), a main PNS, in inhibiting platelet aggregation.

**Methods:** In the *in vitro* study, after incubating platelets with Fc and m-3M3FBS, platelet aggregation was triggered by thrombin, collagen or ADP. Platelet aggregation was measured by aggregometer. Phospholipase Cγ2 (PLCγ2) and protein kinase C (PKC) activities were studied by western blotting. Diacylglycerol (DAG), thromboxane B_2_ (TXB_2_) and 1,4,5-inositol trisphosphate (IP_3_) concentrations were measured by corresponding ELISA kits. Calcium concentrations ([Ca^2+^]) were estimated through the fluorescence intensity emitted from Fluo-4. In the *in vivo* study, thrombus model was induced by FeCl_3_. The effect of Fc on thrombosis was evaluated by measurement of protein content and observation of injured blood vessel.

**Results:** thrombin, collagen and ADP induced platelet aggregation were all suppressed by incubating platelets with Fc. Platelet PLCγ2 and subsequent DAG-PKC-TXA_2_ and IP_3_ were down-regulated by Fc as well. However, the basal [Ca^2+^] in platelet was not altered by Fc. Nevertheless, thrombin triggered activation of PLCγ2 and subsequent DAG-PKC-TXA_2_ and IP_3_-[Ca^2+^] were all abolished by Fc. Fc also attenuated platelet aggregation and PLCγ2 signaling activation induced by PLC activator, m-3M3FBS. In the *in vivo* study, FeCl_3_ induced thrombosis in rat femoral artery was significantly alleviated by administration of Fc.

**Conclusion:** The results above suggested the antiplatelet and antithrombotic effects of Fc are carried out through oppression of PLCγ2 and subsequent DAG-PKC-TXA_2_ and IP_3_-[Ca^2+^]. The present study provided theoretical support for new anti-thrombotic drug exploitation by *Panax notoginseng*.

## Introduction

Platelets are cells in mammal blood, formed from the cytoplasm of bone marrow megakaryocytes (Hartwig and Italiano, 2003). They play important role in a serious of physiological and pathological processes, such as hemostasis, inflammation responses and thrombosis. In normal condition, the main function of platelet is hemostasis. But in the pathological conditions, platelet aggregation was excessively triggered by a series of stimulators in the vascular microenvironment, which may result in thrombosis (Ruggeri and Mendolicchio, 2007). Thrombosis, the foremost precipitating factor for cardiovascular disease, threatened a great many people’s lives during the past decades. Platelet is the primary target for treatment of thrombotic diseases (Mackman, 2008).

Many antiplatelet drugs have been used in clinic for treatment of thrombotic diseases. The dominating defect for the clinically used chemical antiplatelet drugs is the drawback of bleeding, which result from the unidirectional inhibition of platelet aggregation. During antiplatelet therapy, bleeding threatens people’s lives more serious than thrombus itself (Meadows and Bhatt, 2007; Généreux et al., 2015). Although, attempts have been done for discovering new targets and new compounds for antiplatelet drug development, by simply down-regulate platelet aggregation, the defect of bleeding is still ineluctable (Mackman, 2008; Zhang et al., 2015).

*Panax notoginseng*, a plant mainly produced from Yunnan province of China, has been used as a Traditional Chinese Medicine for 100s of years because of its amazing stasis dispersing and hemostatic effects. Traditionally, the medicinal part of the plant is dried root and rhizome (under-ground part), which called “Sanqi.” According to the Traditional Chinese Medicinal theory, thrombosis implies the syndrome of blood stasis. As a result, stasis dispersing and hemostatic drugs are the most appropriate for treatment of thrombosis (Liao, 2000). Sanqi, the best-known stasis dispersing and hemostatic drug, has amazing dual-directional regulatory effect on platelets. Therefore, the distinctive advantage of Sanqi over chemical antiplatelet drugs is removing stasis without bleeding. In addition, Sanqi have a powerful capacity in inhibiting platelet aggregation, which is superior to aspirin (Wang et al., 2016). As a result, Sanqi have good potential to be explored for anti-thrombotic therapy.

However, the price of Sanqi is so high that any drug developed from it would be hardly afforded for most patients. Meanwhile, more than 90% of the over-ground parts of *Panax notoginseng* were abolished, despite the leaves and flowers of *Panax notoginseng* also showed fantastic stasis dispersing and hemostatic effects (Ke et al., 2010). In Yunnan, the over-ground parts of *Panax notoginseng* are more popular for indigenes. The leaves and flowers were made into tea, food and wine. Recent years, since the leaves and flowers of *Panax notoginseng* attracted attentions of researchers, they have been made into nourishment, toothpaste and so on.

Preventive treatment is quite advocated by Traditional Chinese Medicine. It implies preventing disease from occurring and preventing disease from exacerbating (Liang and Yin, 2010). Compared with take drugs, prevent disease by daily food and tea is the easiest way for preventive treatment. Because the price of Sanqi is quite high, utilization of the over-ground part of *Panax notoginseng* instead is a good way for reducing the cost. Pharmacodynamics studies have demonstrated similar functions of *Panax notoginseng* leaves and flowers with Sanqi, including anti-thrombosis, wound healing, anti-hyperlipidemia, anti-depression, anti-inflammation and so on. By drinking *Panax notoginseng* tea made from leaves and flowers, thrombus formation was prevented and the symptoms of thrombotic diseases were relieved a lot. Therefore, food, tea and nourishment made from the leaves and flowers of *Panax notoginseng* will be good resource for thrombotic diseases preventive treatment.

**FIGURE 1:**
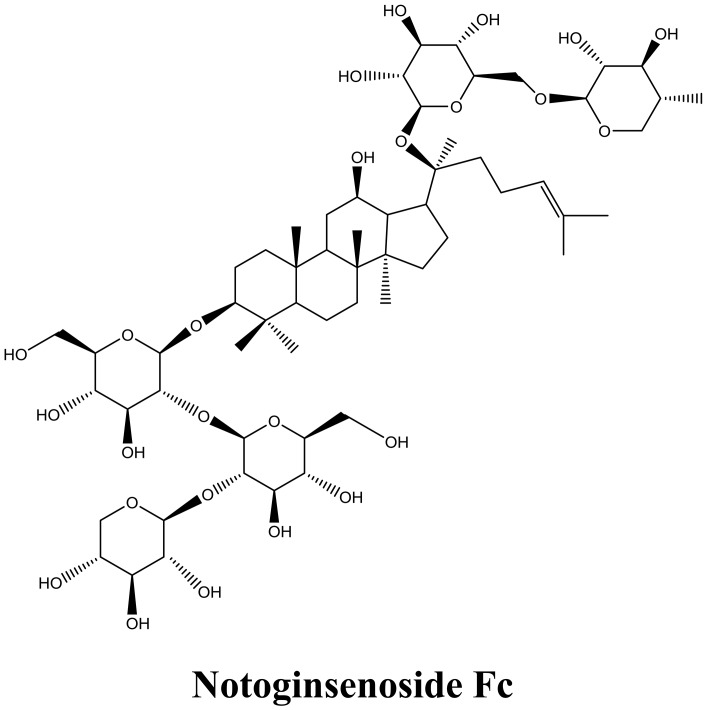
Chemical structure of Notoginsenoside Fc.

The dual-directional regulatory effect of *Panax notoginseng* on platelets should be ascribed to the resourceful PNS (Wang et al., 2004; Yuan et al., 2011; Gao et al., 2014). Until now, over 70 saponins have been isolated from *Panax notoginseng*. Among them, Fc, Ginsenoside Rg1, Rg2, Rg3, Rh2, Re, and Rd were proved capable in inhibiting platelet aggregation. On the other hand, Ft1, Notoginsenoside Fe and protopanaxadiol are effective in promoting platelet aggregation (Gao et al., 2014). Compared with Sanqi, the leaves and flowers are richer in PNS. Furthermore, the content of most effective antiplatelet compound Fc (Figure 1) is the richest in leaves and flowers of *Panax notoginseng*, compared with in roots (Zhou et al., 2017).

Despite the stasis dispersing and hemostatic effect of *Panax notoginseng* is well-known, the mechanism on how the dual-directional regulatory effect been balanced remains to be clarified. Uncovering the molecular mechanisms of main PNS will be quite helpful for clarifying that. Since Fc is the saponin exerts strongest antiplatelet effect among the PNS, the present study aimed at uncovering the mechanism of Fc in inhibiting platelet aggregation.

## Materials and Methods

### Materials

Fc standard was acquired from Shanghai Shifeng Biological Technology CO., LTD. (Shanghai, China). Collagen was purchased from Chrono-log (Havertown, PA, United States). Thrombin, ADP, clopidogrel and m-3M3FBS were obtained from Sigma–Aldrich (St. Louis, MO, United States). Fluo-4 AM indicator was obtained from Invitrogen (Carlsbad, CA, United States). Protease inhibitor and phosphatase inhibitor cocktail tablets were from Roche Diagnostics (Indianapolis, IN, United States). Phospho antibody for PLCγ2, Phospho antibody for PKC substrate and β-actin were purchased from Cell Signaling Technology (Beverly, MA). Immobilon western detection reagents, HRP-conjugated anti-rabbit and anti-mouse IgG were from Genshare Biological (Xi’an, Shaanxi, China). DAG, TXB_2_ and IP_3_ kits were purchased from R&D Systems (Minneapolis, MN, United States). Pierce^TM^ BCA Protein Assay Kit was from Pierce Biotechnology (Rockford, IL, United States). All the chemicals used were purchased from standard suppliers.

### Animals

All animal experiments were approved by the Ethics Committee of Northwest A&F University. Male SD rats (5–6 weeks of age) were purchased from Dossy Experimental Animals CO., LTD. (Xi’an, Shaanxi, China) and acclimated for 1 week before the experiments. The laboratory animal facility was maintained at a constant temperature and humidity with a 12 h light/dark cycle. Food and water were provided *ad libitum*.

### Washed Platelets Preparation

The method for WP preparation was the same as before (Liu et al., 2013). Briefly, blood was withdrawn from the abdominal aorta of rats anesthetized with ether. Acid-citrate-dextrose (66.6 mM citric acid, 85 mM trisodium citrate, 111 mM glucose) was used as anticoagulant (Acid-citrate-dextrose: blood = 1: 6). Then, the blood was centrifuged at 150 × *g* for 10 min. After that, the upper layer platelet rich plasma was centrifuged (150 × *g*) for another 10 min and washed once with washing buffer (138 mM NaCl, 2.8 mM KCl, 0.8 mM MgCl_2_, 0.8 mM Na_2_HPO_4_, 10 mM HEPES, 0.55 mM glucose, 22 mM trisodium citrate, 0.35% BSA, pH 6.5). Finally, the platelet pellets were suspended in suspension buffer (138 mM NaCl, 2.8 mM KCl, 0.8 mM MgCl_2_, 0.8 mM Na_2_HPO_4_, 10 mM HEPES, 5.6 mM glucose, 1 mM CaCl_2_, 0.3% BSA, pH 7.4) to a final concentration of 2 × 10^8^ platelets/ml.

### Platelet Aggregation Study

Platelet aggregation experiments were performed in a LBY-NJ4 platelet aggregometer (Techlink Biomedical). After treated with testing materials, WP aggregation was induced by different stimulators (thrombin, collagen, ADP, or m-3M3FBS). The stimulators were used in the minimal concentrations inducing submaximal aggregation.

### Platelet PLCγ2 and PKC Activity Study

The activity of PLCγ2 and PKC were examined by conventional western blot analysis by suitable antibodies. After treatment with testing materials, platelets were precipitated by centrifugation (12,000 × *g*, 2 min). Then platelets were lysed by lysis buffer (50 μM HEPES, 50 μM NaCl, 50 μM sucrose, 1% Triton X-100, protease inhibitor cocktail, and phosphatase inhibitor cocktail). Protein contents were measured by a Pierce^TM^ BCA Protein Assay Kit from Pierce Biotechnology. The lysates were used as western blotting samples. Western blotting experiment procedures were same as everyone known. The activity of PLCγ2 and PKC were assessed by the phosphorylation of PLCγ2 and a 47 kDa protein of PKC substrate, respectively. Because there is no antibody available for measuring the total protein of PLCγ2 and PKC substrate of rats, β-actin was used as internal reference for protein loaded. Result images were obtained and analyzed with ChemiDoc XRS+ system and Image Lab software (Bio-Rad Laboratories, Hercules, CA, United States).

### Platelet Calcium Concentration ([Ca^2+^]) Study

Intracellular [Ca^2+^] was studied by Fluo-4 AM with a Live Cell Imaging System equipped with TIRF microscope, EMCCD Andor ultra888 and sCMOS Andor zyla4.2Plus (Andor, Belfast, NIR, ENG). Platelets were incubated in washing buffer containing 1 μM Fluo-4 AM and 1% BSA for 30 min. After washing by centrifugation, platelets were suspended in suspension buffer and treated with testing materials. Pictures were taken by the Live Cell Imaging System in a time dependent order. The intracellular [Ca^2+^] were evaluated by analyzing the fluorescence intensity of the pictures.

### Platelet IP_3_, DAG and Thromboxane A_2_ (TXA_2_) Evaluation

The amount of IP_3_, DAG and TXA_2_ were evaluated by IP_3_, DAG and TXB_2_ ELISA assay kits from R&D Systems (Minneapolis, MN, United States), respectively. After incubating WP with indicated materials, reaction was stopped in ice bath. IP_3_, DAG and TXA_2_ content were measured according to the instruction of the test kits.

### *In vivo* Thrombus Study

The method for *in vivo* thrombus study was in accordance with Chinatsu Sakata’s study (Sakata et al., 2017). Rats were grouped randomly and i.p. injected with saline, Fc (50 mg/kg) or clopidogrel (5 mg/kg). The *in vivo* anti-thrombotic effect of Fc was evaluated with a FeCl_3_ arterial thrombosis rat model. Briefly, the rats were anesthetized by i.p. injection of sodium pentobarbital (30 mg/kg). After detach the femoral artery from the surrounding tissues, a filter paper (1 mm × 1 mm) saturated with 20% FeCl_3_ was applied to the artery for 20 min. The injured artery was isolated, observed and photographed under a LECIA M165FC Stereo Microscope (LECIA, Solms, Hesse, GER). Then, the thrombus was isolated gently and dissolved in NaOH (0.5 M). The size of a thrombus was evaluated by the protein content, which was measured by a BCA protein assay kit (Pierce Biotechnology, Rockford, IL, United States).

### Statistical Analyses

Mean and SEM were calculated for all experimental groups. Data were analyzed by One-way Analysis of Variance followed by Dunn’s test, to determine the statistically significant differences. Statistical analyses were performed by SigmaStat Software Ver. 3.5 (Systat Software, San Jose, CA, United States). *P* < 0.05 were considered as statistically significant.

## Results

### Fc Inhibited Platelet Aggregation Induced by Various Stimulators

**FIGURE 2:**
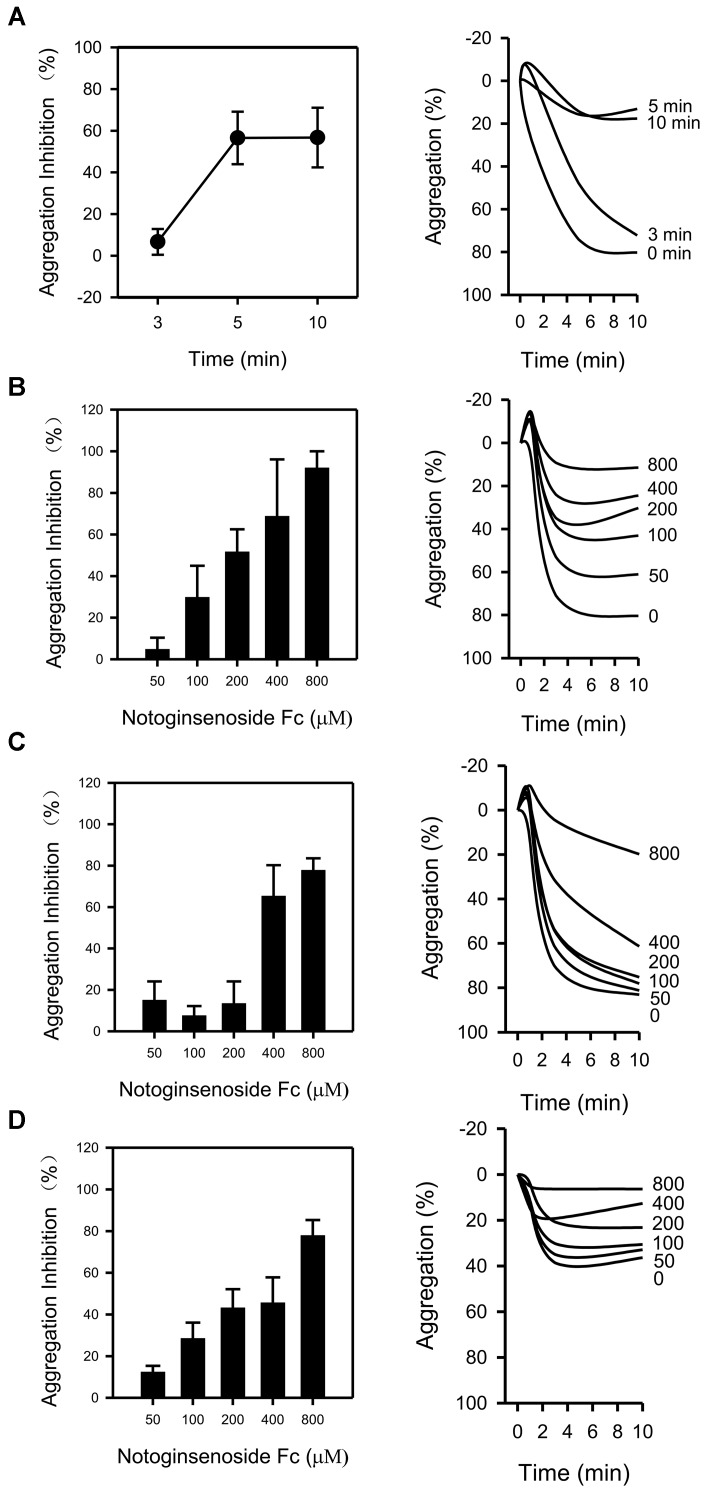
Antiplatelet effect of Fc. **(A)** After incubating WP with Fc (400 μM) for indicated times (3, 5, 10 min), platelet aggregation was induced by thrombin. To determine the dose-dependent antiaggregatory effect of Fc, WP were incubated with indicated concentrations (50, 100, 200, 400, 800 μM) of Fc for 5 min. Platelet aggregation was induced by either thrombin **(B)**, collagen **(C),** or ADP **(D)**. Tracing graphs represent the percentage of platelet aggregation variation after treated with stimulators. Values are mean ± SEM (*n* = 3 for **A**; *n* = 3∼4 for **B** and **C**; *n* = 3∼5 for **D**).

The impact of Fc on platelet aggregation was examined. To determine the appropriate incubating time for the study, several time points (3, 5, and 10 min) were tested against thrombin. The anti-platelet effect of Fc (400 μM) was peaked at 5 min (Figure 2A). As a result, 5 min was used in the following study. After that, the concentration dependent antiplatelet effect for Fc was investigated. Treatment of WP with Fc resulted in proportional suppression of thrombin induced platelet aggregation, with an IC_50_ of 204.38 μM (Figure 2B). Consistent with these results, by pretreatment of WP with increasing concentrations (50, 100, 200, 400, and 800 μM) of Fc, platelet aggregation induced by collagen (Figure 2C) and ADP (Figure 2D) were all inhibited dose-dependently, with IC_50_ of 379.93 and 295.89 μM, respectively. According to these results, Fc can inhibit various stimulators induced platelet aggregation, and most effective to thrombin.

### Fc Down Regulated the PLCγ2 Cascade in Platelet

**FIGURE 3:**
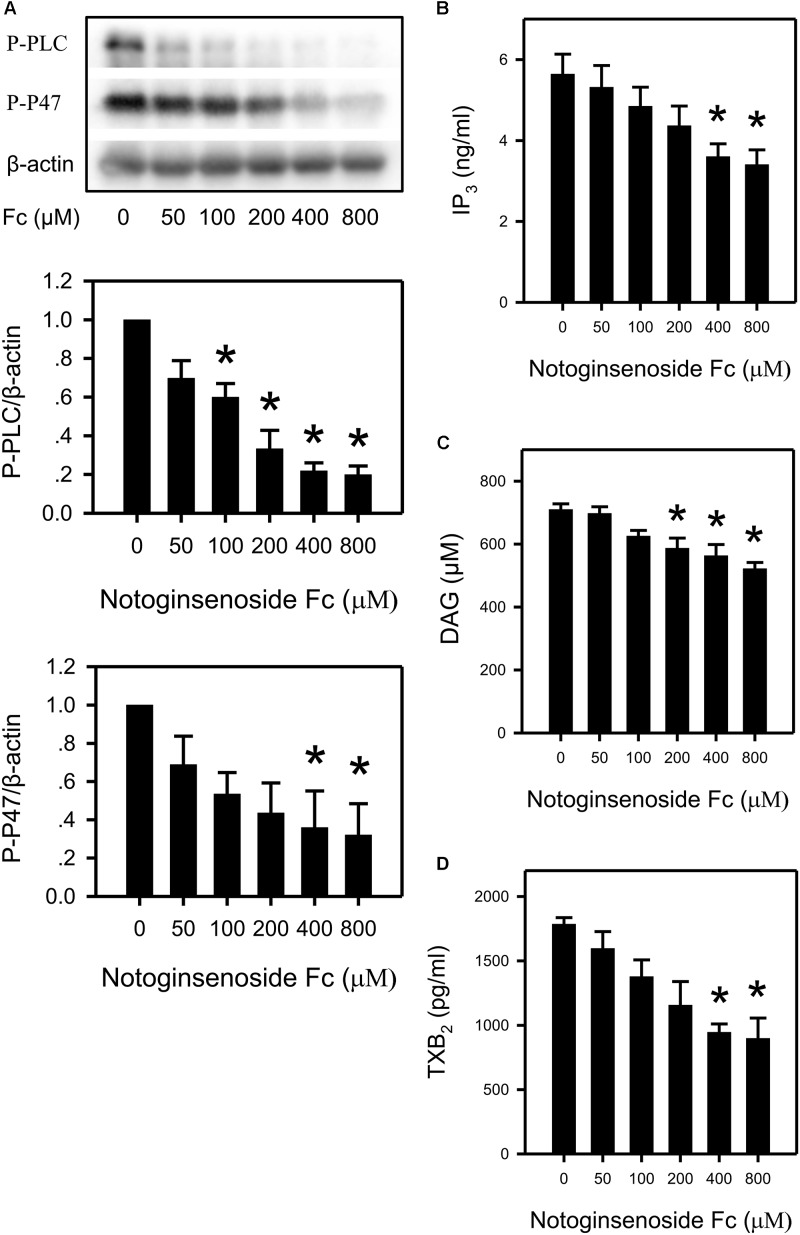
Suppression of platelet PLCγ2 cascade by Fc. **(A)** After incubating WP with different concentrations of Fc, platelet PLCγ2 and PKC activity were evaluated according to the phosphorylation of PLCγ2 and P47. β-actin was used as a loading control. Platelet content of IP_3_, DAG and TXB_2_ were measured by ELISA kits. WP was incubated with the indicated concentrations of Fc, and platelet IP_3_ **(B)**, DAG **(C),** and TXB_2_ **(D)** concentrations were assessed. Values are mean ± SEM (*n* = 3 for **A**; *n* = 5 for **B**; *n* = 4 for **C,D**). ^∗^*P* < 0.05 versus control.

**FIGURE 4:**
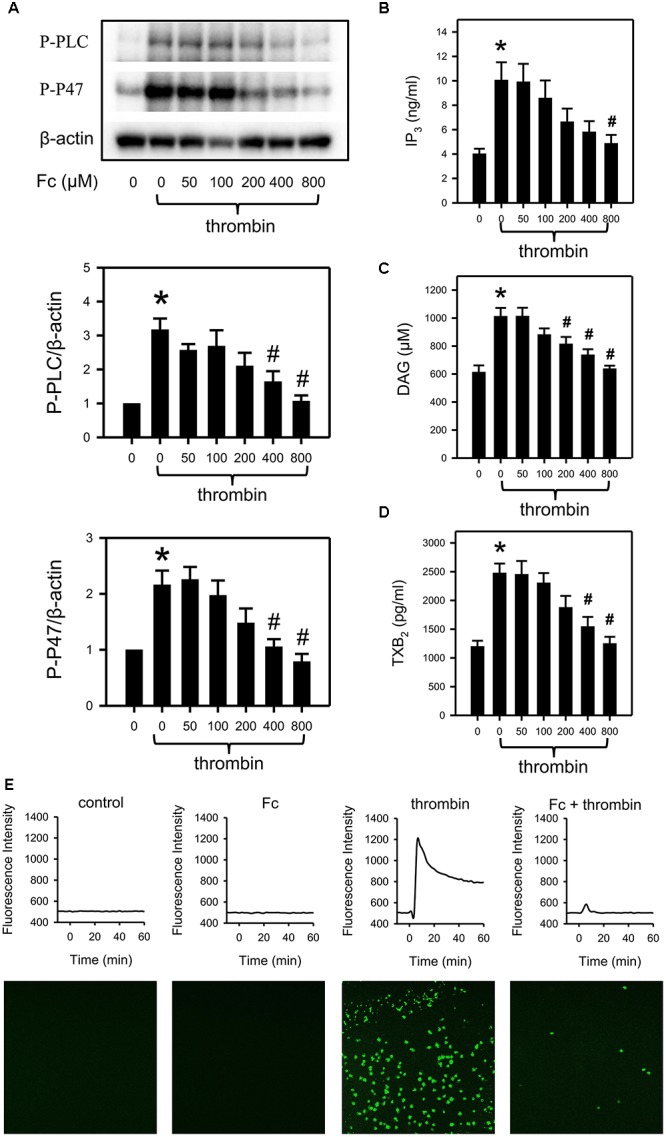
Prevention of thrombin induced activation of PLCγ2 cascade by Fc. After incubating with different concentrations of Fc, WP was treated with thrombin for 3 min. **(A)** The phosphorylation of PLCγ2 and P47 in platelet were measured with western blotting. β-actin was used as a loading control. Platelet IP_3_ **(B)**, DAG **(C)** and TXB_2_ **(D)** concentrations were assessed by corresponding ELISA kits. **(E)** Variation in [Ca^2+^] was assessed by detecting the fluorescence intensity emitted from intracellular Fluo-4. The images are from the peak point of each experiment. Tracings were from representative results in three independent experiments. Values are mean ± SEM (*n* = 6 for **A**; *n* = 4 for **B,C**; *n* = 3 for **D**). ^∗^*P* < 0.05 versus control; ^#^*P* < 0.05 versus thrombin only.

To confirm the involvement of the PLCγ2 cascade in the antiplatelet effect of Fc, the following indexes were measured: P-PLCγ2, DAG, IP_3_, P-P47 (a protein reflect PKC activity), TXB_2_ (a metabolite of TXA_2_) and [Ca^2+^]. By incubating platelets with increasing concentrations of Fc, phosphorylation of PLCγ2 and P47 were reduced dose-dependently (Figure 3A). In addition, platelet IP_3_, DAG and TXB_2_ content were also decreased by Fc in a dose-dependent manner (Figures 3B–D). However, the alteration in intra-platelet [Ca^2+^] was not observed (Figure 4E). This may due to the sensitivity of the fluorescence dye (Fluo-4 AM). Because the basal [Ca^2+^] in resting platelets was too low to discern a further decrease.

### Fc Abolished Thrombin Induced PLCγ2 Cascade Activation

Thrombin induced platelet aggregation was most sensitive to Fc. As a result, we demonstrated the involvement of the PLCγ2 cascade in the anti-platelet effect of Fc against thrombin. In accordance with the previous study, thrombin can activate platelet PLCγ2, P47 (Figure 4A); upregulate IP_3_ (Figure 4B), DAG (Figure 4C), TXB_2_ (Figure 4D), and [Ca^2+^] (Figure 4E). By pre-incubation with increasing concentrations of Fc, thrombin induced platelet aggregation (Figure 2B) and activation of PLCγ2 cascade, including increase in [Ca^2+^], were downregulated dose-dependently (Figure 4). This proved our conjecture that Fc can decrease platelet [Ca^2+^] when it high enough to be detected.

### Fc Attenuated m-3M3FBS Induced Platelet Aggregation and PLCγ2 Cascade Activation

**FIGURE 5:**
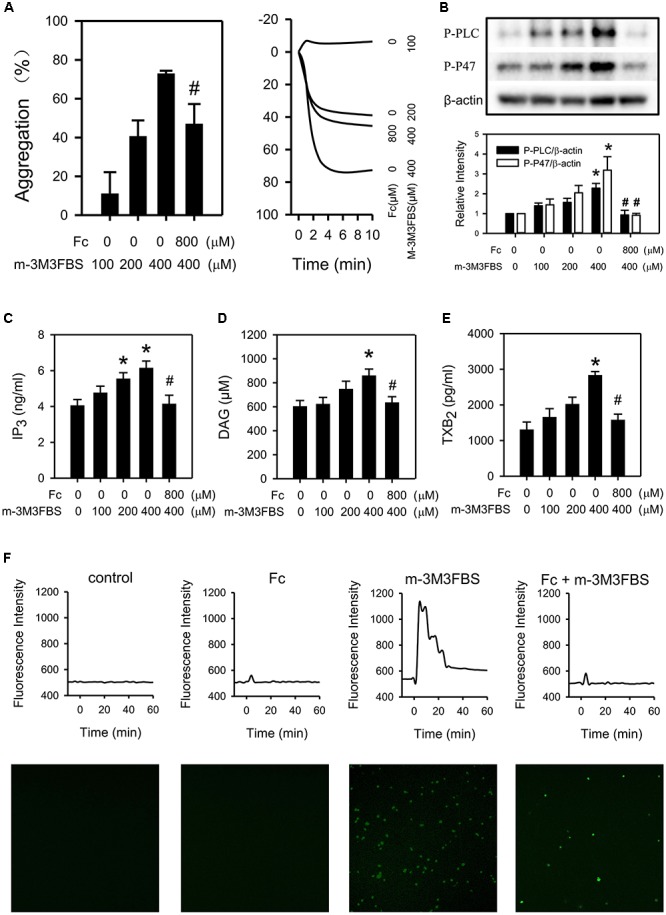
Involvement of PLCγ2 cascade in the antiplatelet effect of Fc. WP were treated with either increasing concentrations of m-3M3FBS (100, 200, 400 μM) or m-3M3FBS (400 μM) followed Fc (800 μM). **(A)** Platelet aggregation was assessed by a 4-channed aggregometer. **(B)** The phosphorylation of PLCγ2 and P47 of platelet were measured with western blotting. β-actin was used as a loading control. Platelet IP_3_ **(C)**, DAG **(D),** and TXB_2_ **(E)** concentrations were assessed by ELISA kits. **(F)** [Ca^2+^] was assessed by the fluorescence intensity from intracellular Fluo-4. The images are from the peak point of each experiment. Tracings were from representative results in three independent experiments. Values are mean ± SEM (*n* = 6∼11 for **A**; *n* = 3∼4 for **B–E**). ^∗^*P* < 0.05 versus control; ^#^*P* < 0.05 versus 400 μM m-3M3FBS only.

M-3M3FBS (100, 200, 400 μM), direct PLC activator, activated PLCγ2 (Figure 5B) and induced platelet aggregation (Figure 5A) in a dose-dependent manner. Meanwhile, m-3M3FBS induced PLCγ2 activation was abolished by Fc (Figure 5B). And platelet aggregation was partially restored by pretreatment of Fc (Figure 5A). This demonstrated Fc can inhibit platelet aggregation through preventing m-3M3FBS induced PLCγ2 activation. In addition, m-3M3FBS increased P-P47, IP_3_, DAG, TXB_2_ and [Ca^2+^] were prevented by Fc as well (Figures 5B–F). The above results proved that Fc inhibit platelet aggregation through oppression the activation of PLCγ2 and subsequent DAG-PKC-TXA_2_ and IP_3_-Ca^2+^.

### Fc Alleviated *in vivo* Thrombus Formation

**FIGURE 6:**
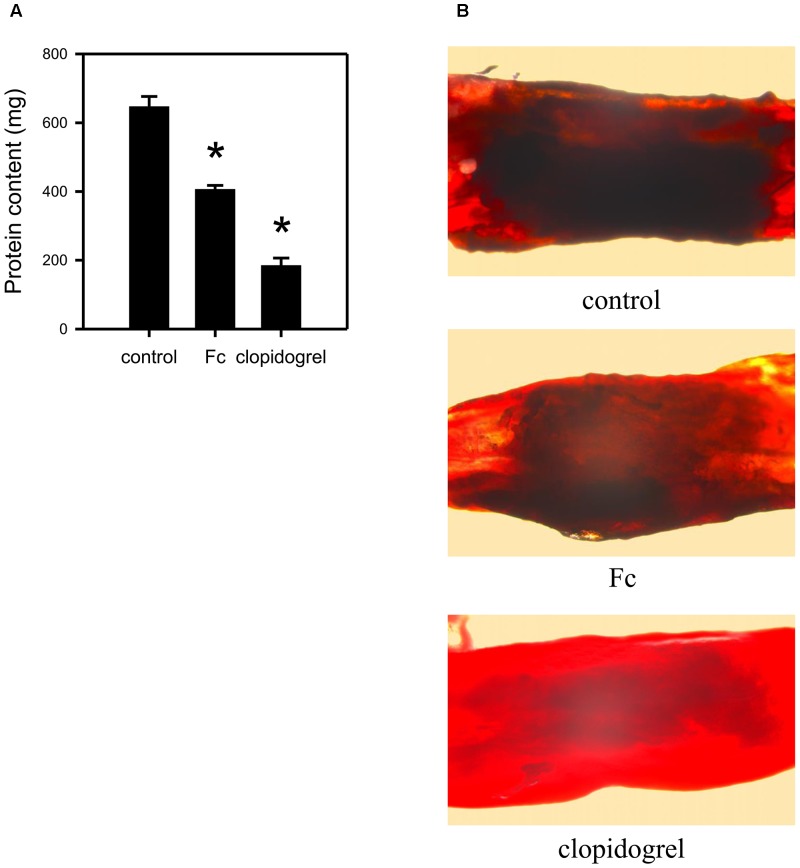
*In vivo* anti-thrombotic effect of Fc. Rats were i.p. injected with saline, Fc or clopidogrel. 2 h later, a filter paper saturated with 20% FeCl_3_ was applied to the isolated femoral artery for 20 min. Then the injured arteries were isolated for observation under microscope and measurement of thrombus protein content. **(A)** The bar graph indicates the protein contents in FeCl_3_ induced thrombus of each group. **(B)** The representative graphs showed the artery condition after injured by FeCl_3_. Values are mean ± SEM (*n* = 6 for control and Fc group; *n* = 3 for clopidogrel group). ^∗^*P* < 0.05 versus control.

To evaluate the *in vivo* anti-thrombotic effect of Fc, a FeCl_3_ thrombosis model was employed. By i.p. injection of Fc, thrombus protein content was decreased from 647.10 ± 72.30 mg to 406.38 ± 28.77 mg, although not as strong as clopidogrel (Figure 6A). FeCl_3_ injured blood vessels were also observed under a microscope. The dark part influencing blood vessel’s transparency was thrombus. Fc administration significantly decreased the thickness of thrombus (Figure 6B). This indicated Fc not only can inhibit *in vitro* platelet aggregation, but also can alleviate *in vivo* thrombus formation.

## Discussion

**FIGURE 7:**
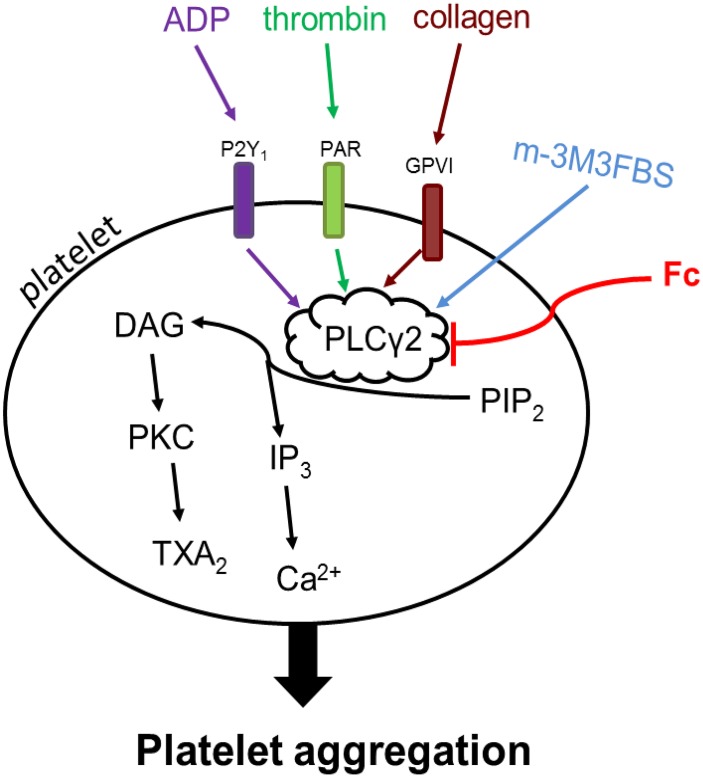
Mechanism of Fc inhibits platelet aggregation. Thrombin, collagen and ADP activate PLCγ2 signaling cascade through PAR, GPVI and P2Y_1_ receptors, respectively. PLCγ2 promotes the hydrolyzation of PIP_2_ to DAG and IP_3_ in platelets. The increase in platelet DAG and IP_3_ subsequently up-regulate PKC activity, TXA_2_ content and [Ca^2+^]. m-3M3FBS triggers activation of PLCγ2 and downstream signaling directly. Fc can abolish platelet aggregation induced by thrombin, collagen, ADP, as well as m-3M3FBS, through preventing the activation of PLCγ2 signaling. Arrows indicate the activation of target molecules or stimulation of production. Blunt line means target inhibition. The difference in color demonstrates different stimulators triggered effects.

Although antiplatelet drugs have been frequently used in the clinic, the problems of bleeding kept push people to develop new drugs for controlling thrombus growth. In this process, a series of targets for antiplatelet drugs have been suggested, most of which are platelet receptors. Collagen, a stimulator released from damaged blood vessel, induces platelet aggregation through activation of glycoprotein VI and integrin α_2_β_1_ receptors. ADP is a platelet activator can be secreted both externally and internally. It activates platelet through P2Y_12_ and P2Y_1_ receptors. Thrombin, the most potent platelet activator, predominately activate platelets by protease activator receptor 1 (PAR_1_) and PAR_4_ (Coughlin, 2005; Leger et al., 2006). The receptors for thrombin, collagen and ADP are different, but there is a well-known pathway involved in all the three stimulators induced platelet aggregation: PLCγ2 activation improves hydrolyzation of PIP_2_ into IP_3_ and DAG, which in turn contribute to Ca^2+^ release, PKC activation and TXA_2_ increase (Si-Tahar et al., 1996; Liu et al., 2005; Ragab et al., 2007; Stegner and Nieswandt, 2011). Thrombin, collagen and ADP trigger PLCγ2 cascade activation through PAR, glycoprotein VI and P2Y_1_ receptors, respectively (Figure 7).

In the present study, Fc inhibited platelet aggregation induced by thrombin, collagen and ADP (Figure 2). Therefore, it is quite possible that PLCγ2 and its downstream signaling are related with antiplatelet effect. Indeed, by measurement of PLCγ2 and PKC activities, IP_3_, DAG, PKC and TXA_2_ concentrations were down-regulated by Fc dose dependently (Figure 3). However, a decrease in [Ca^2+^] was not observed by Fc treatment. This is because the basal level of [Ca^2+^] in platelets was not high enough to see a further decrease. This was proved by testing the influence of Fc on thrombin stimulated platelets. According to the result, PLCγ2 and PKC activities, IP_3_, DAG, PKC, TXA_2_ and Ca^2+^ concentrations raised by thrombin were all abolished by pretreatment of Fc (Figure 4).

Although Fc can restore thrombin induced platelet aggregation and activation of PLCγ2 cascade, the relationship between the anti-platelet effect and PLCγ2 cascade is still not clear yet. Since thrombin induced platelet aggregation is quite complicated, PLCγ2 cascade is only part of it. To exclude the interfuse of other pathway, a direct PLC activator, m-3M3FBS, was employed (Bae et al., 2003). As we expected, PLCγ2 activity in platelets were enhanced by m-3M3FBS dose-dependently. And the increase in light transmission detected by aggregometer usually happens in platelet aggregation were triggered by m-3M3FBS as well. However, m-3M3FBS induced platelet aggregation was only partially restored by Fc (Figure 5). This may because of the non-specific effect of m-3M3FBS. Indeed, platelet aggregation induced by m-3M3FBS through activation of PLC is related with the increase in light transmittance of platelets. But it may also occur during platelet apoptosis (Shcherbina and Remold-O’Donnell, 1999; Stivala et al., 2017). It is reported that m-3M3FBS can affect a serious of apoptosis related proteins: up-regulate pro-apoptotic Bax, down-regulate anti-apoptotic Bcl-2, activate caspase and promote release of cytochrome C (Lee et al., 2005). In addition, PLC was classified into 6 families, including PLCβ, PLCγ, PLCδ, PLCε, PLCζ, and PLCη (Nakamura and Fukami, 2017). Except PLCγ2, PLCβ3 also relates with platelet aggregation (Lee et al., 2014; Pradhan et al., 2017). Therefore, PLCβ3 may also contribute to m-3M3FBS induced platelet aggregation. However, specific activator for PLCγ2 is still not available. After all, m-3M3FBS induced activation of PLCγ2 and subsequent DAG-PKC-TXA_2_ and IP_3_-[Ca^2+^] were almost completely abolished by Fc (Figure 5). Therefore, PLCγ2 cascade indeed responsible to the antiplatelet effect of Fc.

By a single administration of Fc, FeCl_3_ induced thrombosis in rat femoral artery was significantly alleviated (Figure 6). Although the anti-thrombotic potency of Fc is not as strong as clopidogrel, chronic administration may be a safer way for preventive treatment of thrombotic diseases. After all, the key point for new antiplatelet drug development is no longer high potency anymore. As we know, the strong unidirectional antiplatelet effect will result in serious bleeding. Safety is the predominant advantage of *Panax notoginseng* over clinical antiplatelet drugs (Rao et al., 2005; Bittl et al., 2016; Moon et al., 2018). By treatment of *Panax notoginseng*, when Fc exerts its effect on platelets, other saponins may provide complementary or eliminatory assistance. For instance, Ft1, another saponin from over-ground part of *Panax notoginseng*, was proved to have effect in promoting platelet aggregation by activating P2Y_12_ receptors (Gao et al., 2014). Therefore, a hypothesis was put forward: as main saponins in over-ground part of *Panax notoginseng*, Ft1 and Fc may be the predominant compositions for the dual-directional regulatory effect on platelet. To clarify the way to balance the dual-directional regulatory effect and comprehensive impact of them on platelet and thrombus, further research is required.

The effect of PNS on inhibiting platelet aggregation has been mentioned in many studies. PNS can inhibit platelet aggregation through PPAR-γ/PI3K/Akt/eNOS pathway (Shen et al., 2017), [Ca^2+^], ERK_2_/p38 (Qi et al., 2016), COX (Wang et al., 2016), FAK, NF-κB (Yuan et al., 2011). Whereas, most of them didn’t mention any single saponin, but only investigated the complex PNS. Which single saponin stimulated the above signals is still unknown. The hemostatic effect of *Panax notoginseng* is well known, but no systematic study about it yet. Gao et al. (2014) screened several saponins and conclude only Notoginsenoside Ft1, Notoginsenoside Fe and protopanaxadiol are effective in increase ADP induced platelet aggregation. Among them, only Ft1 was demonstrated to activate platelet through P2Y_12_ receptors. The effect of Notoginsenoside Fe and protopanaxadiol were not further studied (Gao et al., 2014). Based on these studies, it is hard to elaborate the mechanism of the dual-directional regulatory effect of *Panax notoginseng*. Therefore, our group is trying to investigate the effect and mechanism of more single saponins on platelet. After that, the dual-directional effect of *Panax notoginseng* can be clarified.

The present study demonstrated the mechanism of the antiplatelet effect of Fc, a main functional saponin in leaves and flowers of *Panax notoginseng*. This can provide theoretical basis for utilization of over-ground part of the plant. Attentions should be paid to the dual-directional regulatory effect of *Panax notoginseng* on platelets, which may be a new perspective for thrombotic disease treatment.

## Ethics Statement

This study was carried out in accordance with the recommendations of the guideline for the use of Laboratory Animals, the Ethics Committee of Northwest A&F University. The protocol was approved by the Ethics Committee of Northwest A&F University. Male SD rats (5–6 weeks of age) were purchased from Dossy Experimental Animals Co., Ltd. (Xi’an, Shaanxi, China) and acclimated for 1 week before the experiments. The laboratory animal facility was maintained at a constant temperature and humidity with a 12 h light/dark cycle. Food and water were provided *ad libitum*. After acclimation, rats were randomly divided into 3 groups, and i.p. injected with saline, Fc (50 mg/kg) or clopidogrel (5 mg/kg), respectively. Two hours after i.p. injection, the rats were anesthetized by i.p. injection of sodium pentobarbital (30 mg/kg). After detach the femoral artery from the surrounding tissues, a filter paper (1 mm × 1 mm) saturated with 20% FeCl3 was applied to the artery for 20 min. The injured artery was isolated, observed and photographed under Microscope. Then, the thrombus was isolated gently and dissolved in NaOH (0.5 M). The size of a thrombus was evaluated by the protein content. The rats were sacrificed with diethyl ether after experiment.

## Author Contributions

YqL, LC, and XS designed the research. YqL, TL, ZL, YyL, and TH performed the experiments. YqL, KD, WZ, YF, and WM analyzed the data. YqL, LC, and KD wrote the manuscript.

## Conflict of Interest Statement

The authors declare that the research was conducted in the absence of any commercial or financial relationships that could be construed as a potential conflict of interest.

## Abbreviations

[Ca^2+^]: calcium concentration
DAG: diacylglycerol
Fc: Notoginsenoside Fc
Ft1: notoginsenoside Ft1
IP_3,_ 1,4,5-inositol trisphosphate; PAR: protease activator receptor
PIP_2,_ phosphatidylinositol 4,5-bisphosphate; PKC: protein kinase C
PLCγ2: phospholipase Cγ2
PNS: *Panax notoginseng* saponins
TXA_2,_: thromboxane A_2_
TXB_2,_: thromboxane B_2_
WP: washed platelets
